# COVID-19 Vaccine Booster Strategies for Omicron SARS-CoV-2 Variant: Effectiveness and Future Prospects

**DOI:** 10.3390/vaccines10081223

**Published:** 2022-07-30

**Authors:** Dorota Zarębska-Michaluk, Chenlin Hu, Michał Brzdęk, Robert Flisiak, Piotr Rzymski

**Affiliations:** 1 Department of Infectious Diseases, Jan Kochanowski University, 25-369 Kielce, Poland; dorota1010@tlen.pl (D.Z.-M.); michal.brzdek@gmail.com (M.B.); 2 College of Pharmacy, University of Houston, Houston, TX 77204, USA; chu7@uh.edu; 3 Department of Infectious Diseases and Hepatology, Medical University of Białystok, 15-540 Białystok, Poland; robert.flisiak1@gmail.com; 4 Department of Environmental Medicine, Poznan University of Medical Sciences, 60-806 Poznan, Poland; 5 Integrated Science Association (ISA), Universal Scientific Education and Research Network (USERN), 60-806 Poznań, Poland

**Keywords:** COVID-19, Omicron, booster, vaccine effectiveness

## Abstract

In the light of the lack of authorized COVID-19 vaccines adapted to the Omicron variant lineage, the administration of the first and second booster dose is recommended. It remains important to monitor the efficacy of such an approach in order to inform future preventive strategies. The present paper summarizes the research progress on the effectiveness of the first and second booster doses of COVID-19. It also discusses the potential approach in vaccination strategies that could be undertaken to maintain high levels of protection during the waves of SARS-CoV-2 infections. Although this approach can be based, with some shortcomings, on the first-generation vaccines, other vaccination strategies should be explored, including developing multiple antigen-based (multivariant-adapted) booster doses with enhanced durability of immune protection, e.g., through optimization of the half-life of generated antibodies.

## 1. Introduction

The Omicron lineage of SARS-CoV-2 (B.1.1.529) was first detected in November 2021 in Botswana and South Africa [1]. It accumulated over 50 sense mutations in the genome, among which approximately thirty induced amino-acid changes in the spike protein, with ten concerning the receptor-binding domain [2]. Such a high number of mutations led to substantial evasion of naturally-acquired and vaccine-induced immunity, ultimately resulting in increased transmissibility [3,4]. Consequently, within several weeks Omicron variant became a predominant SARS-CoV-2 variant circulating globally [5]. As shown, a key feature of Omicron behind this rapid spread was not an increased viral load than for the preceding Delta variant ([6] or higher affinity to the ACE2 receptor [7], but substantial evasion of naturally-acquired and vaccine-induced neutralizing antibodies [8,9]. This had raised concerns over the efficacy of the authorized vaccines designed in 2020, long before the emergence of Omicron, which were using spike protein without the critical changes found in Omicron [10]. The main vaccines used worldwide included mRNA-based vaccines BNT162b2 (BioNTech/Pfizer, Mainz, Germany/New York, NY, USA) and mRNA-1273 (Moderna Therapeutics, Cambridge, MA, USA), adenoviral vector vaccines AZD1222 (Oxford/AstraZeneca, UK/Sweden) and Ad26.COV2.S (Janssen/Johnson & Johnson, Beerse, Belgium/New Brunswick, NJ, USA) and inactivated vaccine CoronaVac (Sinovac, Beijing, China) [10].

On the other hand, the first-generation vaccines remained effective against other variants, such as Delta, which were also characterized by the mutated spike protein [11,12,13]. However, to maintain high levels of protection, a booster strategy has been implemented [14,15]. This decision was also motivated by the observations of the gradual decrease of the antibody levels within several weeks from the last dose [16,17], which increased the odds of breakthrough infections [18].

Since the Omicron-adapted vaccines remain under clinical development [19,20,21], the administration of a booster dose, and recently also a second booster dose, based on the original version of vaccines (the majority of which are mRNA vaccines) have been thus recommended to curb the globally increasing transmission of Omicron variant. Therefore, it remains important to monitor the effect of booster strategies during the dominance of the Omicron lineage and, if necessary, offer some potential future vaccination strategies. It is imperative given that antibody levels following administration of a booster dose also tend to decrease, usually within 3–4 months, while the Omicron variant is, contrary to Delta lineage, better adapted to reduce the recognition of spike protein by T-cells [22]. The evaluation of booster strategies can be performed by assessment of the vaccine’s efficacy which is measured in a controlled clinical trial (by comparing the outcome in vaccinated individuals to those receiving a placebo and calculation of the relative risk), or by evaluation of the vaccine’s effectiveness, which is measured in the real-world studies and allows us to understand how well the vaccines work to protect communities as a whole.

In the present paper, we update the research progress on the effects of the first and second booster doses of COVID-19 and discuss the potential modifications to vaccination strategies to maintain high levels of protection during the future waves of SARS-CoV-2 infections caused by Omicron variant or novel viral lineage that may emerge.

## 2. The Increased Omicron-Neutralizing Activity of the Booster (Three Doses) Vaccination

The objective of this section was to review the effect that booster COVID-19 vaccination can have on immune responses in the context of the Omicron variant. Due to the substantial genetic mutation in its spike protein [1], Omicron variants exhibit an increased escape from vaccine-induced neutralizing antibodies. This is because the COVID-19 vaccines authorized in 2020/2021 were based on the spike protein of the SARS-CoV-2 version that emerged in Wuhan, China, in late 2019. Subsequently, they poorly match the Omicron lineage. Neutralization of Omicron could even be undetectable in the selected individuals who completed only the primary vaccination course [23]. However, the mRNA vaccine booster elicited neutralizing protection against the Omicron variant [23,24,25,26,27,28]. The mRNA vaccine BNT162b2 (Pfizer) booster substantially increased the neutralizing antibody titers against two major Omicron sub-lineages (BA.1 and BA.2), whereas the corresponding neutralizing antibody titers were lower six months after the initial vaccination [29]. Similarly, Pedersen et al. documented that the serums of participants receiving the homologous BNT162b2 booster can neutralize both Omicron BA.1 and BA.2 isolates [30]. Nemet et al. also reported a substantially increased geometric Omicron-neutralizing titer in the healthy participants receiving the BNT162b2 booster, compared to the low geometric Omicron-neutralizing titer value (in the participants receiving two-dose BNT162b2 vaccine [31]. In a prospective observational study in older nursing home residents (68–98 years old) in France, the BNT162b2 booster dose increased the mean of Omicron-specific neutralizing titers by nearly 2 fold in the SARS-CoV-2-naïve and COVID-19 recovered residents three-months after administration, respectively. The percentage of residents with detectable Omicron-neutralizing antibodies also substantially increased to >80% [32]. Canaday et al. examined the effect of boosting with BNT162b2 mRNA vaccine on humoral immunity and Omicron-specific neutralizing activity among nursing home residents and healthcare workers in the USA, and it was found that the booster administration increased the proportion of participants with detectable Omicron-neutralizations range to 86%, whereas only 28% of participants after full-dose vaccination had the detectable Omicron-specific neutralization activity [33]. Pajon et al. reported that the geometric mean titer level against Omicron variants after one month of mRNA-1273 booster was 20-fold higher than after a month of the primary vaccination course with two-dose mRNA-1273 but rapidly declined by 85% after six months [34]. The inactivated virus vaccine booster substantially increased the neutralizing antibody responses to Omicron lineage variants in a cohort study on the adaptive responses of CoronaVac booster in the healthcare professional receiving the CoronaVac booster nine months after two-dose CoronaVac vaccination. Over 55% of subjects demonstrated the Omicron-neutralizing activities after the boosting dose-the geometric mean titer increased by 2 fold [35]. Moreover, a third dose of the protein subunit vaccine MVC-COV1901 (Medigen Vaccine Biologics Zhubei, Hsinchu County, Taiwan), containing an adjuvanted stable prefusion spike protein of SARS-CoV-2 (similarly to spike protein encoded by the mRNA vaccines), was reported to improve the neutralizing capacity against Omicron [36]. All these studies highlighted the increased neutralizing activity of booster vaccination against Omicron variant despite being lower than that against wildtype variants, as well as the rapid decline over the following months. Moreover, data from studies in non-human primates indicate that boost administration induces comparable immunity and protection against the Omicron variant shortly after administration, regardless of whether an ancestral spike-matched vaccine or Omicron-matched vaccine was used [37].

Additionally, the Omicron-neutralizing activity of the heterologous booster vaccination was also studied. In a subject-blinded, randomized-controlled trial to assess the immunogenicity and safety of heterologous booster COVID-19 vaccination compared with a homologous booster regimen in Singapore, heterologous mRNA-1273 booster vaccination (following a primary course of vaccination with BNT162b2) induced a more robust neutralizing response against the Omicron variant in older individuals compared with homologous BNT62b2 [38]. Wang et al. showed that both the homologous inactivated whole-virion vaccine BBIBP-CorV (Sinopharm, Beijing, China) booster and the heterologous booster with protein subunit vaccine ZF2001 (Anhui Zhifei Longcom Biopharmaceuticals, Hefei, China) considerably increased neutralization titers for Omicron variant [39]. In a phase 4, a non-inferiority, single-blind, randomized study in Brazil, the Omicron-neutralizing capacity was found to substantially increase in the serum samples of the participants who received the primary vaccination regimen of the inactivated virus vaccine CoronaVac and further received a third homologous dose of CoronaVac. However, those who received a third heterologous dose of Ad26.COV2.S, BNT162b2, or AZD1222 had a higher neutralizing response than those who received the homologous booster [40]. Therefore, heterologous immunization with inactivated vaccine followed by mRNA-booster elicits strong immunity against the SARS-CoV-2 Omicron variant [41]. For the population receiving CoronaVac as the primary vaccine, the heterologous booster with other types of vaccines (e.g., mRNA vaccine) can be considered [42]. The summary of reviewed studies in this section is provided in Table 1.

## 3. The Increased Effectiveness of the Booster Vaccination (Three Doses) against the Omicron Variant

This section aimed to review the effectiveness of booster COVID-19 vaccination, based on non-Omicron adapted vaccines, in protection against the Omicron variant of SARS-CoV-2. The increased vaccine effectiveness of the booster against Omicron-associated infection has been widely reported. In a prospective observational study including data of 11,690 adults across 21 hospitals in the USA, the effectiveness of mRNA vaccination (BNT162b2 and mRNA-1273) to prevent Delta variant-associated hospitalization was 85% and 94% for two doses and three doses (booster), respectively, whereas the effectiveness of the mRNA vaccination against Omicron variant was 65% and 86% for two doses and booster, respectively [43]. Tartof et al. showed that the nine-month effectiveness of two doses of the BNT162b2 vaccine against hospital admission due to Omicron infection was 41% while against emergency department admission was 31%. However, after three doses, the effectiveness of BNT162b2 against hospital admission due to the Omicron variant was 85% at <3 months but declined to 55% after ≥3 months [44]. In a test-negative case-control design research in England, the increased vaccine effectiveness (67.2%, at 2-4 weeks) of the BNT162b2 (Pfizer) booster dose against the Omicron variant was observed among patients who received full-dose BNT162b2 vaccination, and the vaccine effectiveness declined to 45.7% at ≥10 weeks [45]. Likewise, in a large, test-negative case-control study in California, USA, the increased vaccine effectiveness (71.6%) of the mRNA-1273 vaccine booster against Omicron infection was observed in participants at 14–60 days after the booster. However, the booster vaccine effectiveness decreased to 47.4% at >60 days [46]. Šmíd et al. showed that the vaccine effectiveness of a recent booster in the Czech Republic increased to 56% against Omicron infection and increased to 87% against Omicron hospitalization, whereas the vaccine effectiveness of the primary course of vaccination against Omicron hospitalization was 45% [47]. Ferdinands et al. showed that during the Omicron period, the booster vaccine effectiveness against COVID-19-associated emergency department/urgent care visits and hospitalizations increased to 87% and 91%, respectively, during the first two months after the booster, but declined to 66% and 78%, respectively, by the fourth month after the booster [48]. Butt et al. showed that vaccine effectiveness of booster vaccination relative to primary vaccination series (relative vaccine effectiveness) was 19%, 52%, and 83% for confirmed infection, hospitalization, admission to intensive care unit, or death, respectively. Despite the relatively low vaccine effectiveness of mRNA booster vaccine dose against the Omicron infection, the booster administration provides substantial protection from hospitalization and performs well in preventing the most severe/critical forms of the disease [49]. Modes et al. showed that the likelihoods of both intensive care unit admission and death during the Omicron-predominant period were lowest among adults who had received a booster dose, highlighting the importance of booster vaccination [50]. Additionally, Plumb et al. also reported that during the Omicron-predominant period, the estimated vaccine effectiveness against reinfection-associated hospitalization increased to 68% after a booster dose from 35% after two-dose primary vaccination [51].

Although the primary course of vaccination was based on various vaccines, the booster strategies were mostly based on mRNA vaccinations [10]. As a result, some individuals were receiving heterologous booster vaccines making it necessary to understand the safety, immunogenicity, and efficacy of such an approach [52,53,54,55]. In relation to the Omicron variant, Accorsi et al. showed that all three studied booster regimens (Ad26.COV2.S/Ad26.COV2.S, Ad26.COV2.S/mRNA, and mRNA/mRNA/mRNA) protected against symptomatic infection, while the highest vaccine effectiveness occurred in the regimens (Ad26.COV2.S/mRNA and mRNA/mRNA/mRNA) that included a booster dose of an mRNA vaccine [56]. The lowest vaccine effectiveness was observed with the homologous Ad26.COV2.S/Ad26.COV2.S booster [56]. In the retrospective cohort studies in Qatar, Abu–Raddad et al. reported decreased cumulative incidences (2.4% and 1.0%) of symptomatic Omicron infection in the homologous BNT162b2 and mRNA-1273 booster cohort, respectively, compared to those (4.5% and 1.9%) of corresponding non-booster cohorts [57]. The effectiveness of the BNT162b2 and mRNA-1273 booster against symptomatic Omicron infection was estimated to be 49.4% and 47.3%, respectively [57]. In a test-negative design using consolidated national administrative data in Malaysia, Suah et al. compared the effectiveness of homologous and heterologous BNT162b2, CoronaVac, and AZD1222 booster vaccination against Delta and Omicron infection (heterologous booster vaccinations: (i) 2 × CoronaVac + 1 × BNT162b, (ii) 2 × CoronaVac + 1 × AZD122, and (iii) 2 × AZD122 + 1 × BNT162b), boosting was associated with the higher adjusted marginal effectiveness values, and a BNT162b2 booster was recommended for the subjects receiving the primary regimen with inactivated and vectored vaccines [58]. Analyzing 80,287 emergency department/urgent care visits and 25,244 hospitalizations among adults across 10 US states during the Omicron predominance period (16 December 2021–7 March 2022), Natarajan et al. reported that the effectiveness of heterologous booster practice (1 × Ad26.COV2.S/1 × mRNA dose) against emergency department/urgent care visits and hospitalization (79% and 78%, respectively) were higher than those (54% and 67%, respectively) of the homologous Ad26.COV2.S booster vaccination (2 × Ad26.COV2.S). However, they were lower than those of 3 × mRNA doses (83% and 90%, respectively). Therefore, the vaccinees receiving Janssen primary vaccine should preferentially receive the mRNA vaccine booster to improve the protection against Omicron [59]. All these studies demonstrated the increased vaccine effectiveness of the existing vaccine booster against the Omicron variant, especially the heterologous booster vaccination, compared to primary vaccination with the non-mRNA vaccine. Therefore, the mRNA vaccine booster was recommended for the individuals who previously received the non-mRNA primary vaccination.

All in all, the administration of a booster dose of the first-generation COVID-19 vaccines increases the protection level against Omicron infection and hospitalization. However, this protection declined compared to that against other SARS-CoV-2 variants. As shown through clinical trials and real-world observations, the efficacy of the primary course of vaccination against variants circulating in 2020 and the first half of 2021 was higher than the efficacy against Omicron lineage after a booster administration [11,18,60,61,62,63,64,65]. Moreover, the level of protection from infection and hospitalization tends to decrease three-four months after administration [44,48,65,66]. As recently summarized by Higdon et al., the effectiveness of booster vaccination against Omicron one month after its administration was higher for all outcomes compared to the effectiveness of the primary vaccine course against the same variant. However, as estimated using random-effects meta-regression, effectiveness against symptomatic infection decreased by 24% within the first four months and by 5% in relation to protection from hospitalization, with further decrease projected by six months [44]. The summary of reviewed studies in this section is provided in Table 2.

## 4. Fourth Dose–What Is the Evidence So Far?

This section’s objective was to review the studies reporting on the effect of administration of an additional booster dose (second booster) of the COVID-19 vaccine not adapted specifically to match Omicron lineage. On 29 March 2022, in response to growing concerns about a rapid decline in the Omicron-neutralizing activity of the booster, the Food and Drug Administration (FDA) authorized a second booster dose of either BNT162b2 or mRNA-1273 vaccines for older persons and immunocompromised individuals who are considered high-risk populations for severe COVID-19. This additional shot is the fourth vaccine dose for immunocompetent people, and for patients with a weakened immune system, it is the fifth due to the three-dose primary vaccination. FDA authorization was followed immediately by a recommendation from the Centers for Disease Control and Prevention to allow eligible persons to receive a second booster dose. Both institutions decided without consulting their vaccine advisory committees, which is an unusual procedure, explaining it as the growing threat of a wave of BA.2 omicron variant infections. A second booster dose of BNT162b2 or mRNA-1273 was recommended for persons 50 years of age and older at least four months after the last shot of any already-approved COVID-19 vaccine. In addition, the BNT162b2 vaccine may be administered on the same schedule to individuals 12 years of age and older following solid organ transplantation or with an equivalent degree of immunodeficiency. Recommendations for the fourth dose of the vaccine were based on published findings from Israel suggesting better protection against the severe course of the disease and no safety issues [67,68]. Israel became the first country worldwide to begin using the fourth dose of the BNT162b2 vaccine in January 2022. According to the regulations, this was offered to health care professionals and adults over 60 years [69].

The effect of additional booster dose administration based on the first-generation COVID-19 vaccines was already a subject of selected real-world studies and prospective, open-label, non-randomized studies (Table 3) [67,68,70]. The latter evaluated both mRNA vaccine formulations, BNT162b2 and mRNA-1273, administered to Israeli medical staff vaccinated with the third dose of BNT162b2 at least four months earlier with IgG antibody levels equal to or lower than 700 BAU/mL [67]. The analysis included 1050 health care workers, 154 persons received the fourth dose of the BNT162b2 vaccine, and another 120 received the mRNA-1273 vaccine one week later. The control group consisted of the age-matched individuals meeting the same eligibility criteria, two for each vaccine recipient. All participants were screened weekly for SARS-CoV-2 infection by PCR testing. Both formulations induced a 9–10-fold increase in IgG antibodies against the SARS-CoV-2 receptor-binding domain and neutralizing antibody titers within two weeks after vaccination. There was also an 8–10-fold increase in live neutralization against the Omicron and other variant strains with titer restoration to the peak after the third dose of BNT162b2n. Compared to controls, the efficacy against SARS-CoV-2 infection compared to controls was 30% and 11% for BNT162b2 and mRNA-1273, respectively. Although sequencing of the infecting virus was not performed, the omicron variant accounted for 100% of the isolates typed during the study period, so it can be assumed that it was responsible for breakthrough infections. The higher protection was documented against symptomatic COVID-19, 43% and 31% for BNT162b2 and mRNA-1273, respectively, compared to controls. However, the authors emphasized that within the wide confidence intervals of the estimates, the vaccine’s effectiveness in protecting against symptomatic disease did not exceed 65%. Therefore, the incidence of breakthrough infections, mostly mild, was high. The study also demonstrated that the fourth dose of mRNA vaccine was safe despite triggering mild systemic and local symptoms in most recipients.

Although in the study by Regev–Yochay et al. [67], including only healthcare workers, the primary endpoints were immunogenicity of the fourth dose of mRNA vaccines and their safety, and the secondary endpoint was efficacy, the studies published later focused on older adults and evaluated only the clinical effects of administering the fourth dose of BNT162b2 vaccine [68,70].

For the purpose of the analysis conducted by Bar-On et al., data were obtained from the Israeli Ministry of Health database between 10 January through 2 March 2022, covering the period of the dominance of the omicron variant, B.1.1.529. The records of 1,252,331 individuals over 60 years who were eligible for the fourth dose of BNT162b2 were analyzed. The risk of both confirmed SARS-CoV-2 infection and severe COVID-19 was assessed in the patients who received the fourth dose compared to the population after three doses of the vaccine. The incidence of confirmed SARS-CoV-2 infection was lower with the fourth dose by a factor of 2.0 compared to only three doses and by a factor of 1.8 compared to the internal control group consisting of patients 3–5 days after the fourth dose of BNT162b2.

The four-dose group achieved a lower rate of severe COVID-19 by a factor of 3.5 and 2.3 compared to that in the three-dose group and the internal control group, respectively. In addition, protection against confirmed SARS-CoV-2 infection waned with time, whereas the prevention of severe disease was stable over six weeks. However, the analysis did not include other endpoints, such as the risk of hospitalization and death. Another limitation of the study was that it did not consider the impact of comorbidities, which are independent risk factors for severe COVID-19.

The assessment of the efficacy of the fourth dose of the BNT162b2 vaccine, considering the risk of confirmed SARS-CoV-2 infection, symptomatic and severe disease, hospitalization, and death due to COVID-19, was performed by Magen et al. [70]. This observational study from Israel was conducted among individuals aged ≥60 from 3 January through 18 February 2022. Individuals who previously had not been infected with SARS-CoV-2 and received a fourth dose of the vaccine were compared with the population vaccinated with a third dose at least four months earlier by individually selecting subjects based on sociodemographic and clinical variables. Data of 182,122 matched pairs of patients from medical records of the largest Israeli health care organization was included in the analysis. Relative vaccine efficacy assessed 14 to 30 days after the fourth dose for protection against SARS-CoV-2 infection, symptomatic COVID-19, hospitalization, severe disease, and death was 52%, 61%, 72%, 64%, and 76%, respectively. A limitation of this study was that the follow-up time was too short to assess the long-term effect of the fourth dose.

In summary, the existing studies demonstrate that administering the second booster dose increases the protection levels against all outcomes related to the Omicron variant (Table 3). However, it is also clear that this effectiveness is reduced due to antigen mismatch between vaccines and circulating viral variants. This mismatch also increased with the emergence of BA.4 and BA.5 subvariants [71] and will likely continue due to the evolution of SARS-CoV-2. The use of first-generation vaccines, which are readily available, can temporarily improve protection levels–a necessity especially during the predicted future waves of SARS-CoV-2 infections (e.g., during the autumn-winter season in the USA and Europe [72,73]).

## 5. Conclusions

**Figure 1:**
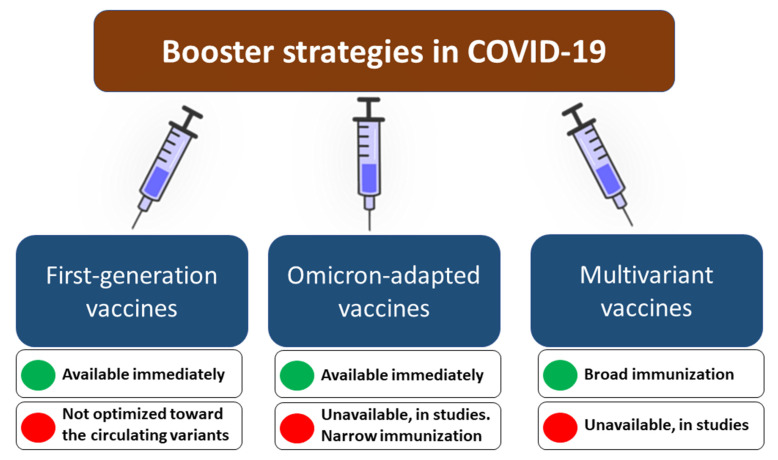
The potential booster vaccination strategies in COVID-19 to maintain high protection levels during future SARS-CoV-2 infection waves with their advantages and disadvantages.

Although the COVID-19 vaccination remains pivotal in decreasing the overall COVID-19 burden and suppressing SARS-CoV-2 evolution [74,75], it is essential to recognize that receiving the primary course vaccination does not protect sufficiently against infection with the Omicron lineage variant. The booster vaccination increases Omicron-neutralizing activity, thereby improving the effectiveness of preventing Omicron-associated infection, symptomatic and severe disease, hospitalization, and death. However, this effect wanes over time due to a gradual decrease in antibody levels three to four months after booster administration, while the vaccine-induced T-cells responses to spike protein of the Omicron variant are lower than to preceding SARS-CoV-2 variants. We outline the potential strategies that could be considered in order to maintain high levels of protection during future waves of SARS-CoV-2 (Figure 1). One should however bear in mind that in any case, the risk/benefit ratio must be analyzed before recommending the booster vaccination [52,76,77,78].

1. Offering an additional (second) booster dose of the first generation before the expected wave. This approach has potential limitations in the long-term effectiveness since first-generation vaccines are not adapted to a heavily mutated spike protein of the Omicron variant, while novel sublineages of this variant (i.e., BA4, BA5), with high immune evasion, are emerging [71]. The necessity for repeated vaccinations may be met with increasing unwillingness and hesitancy–the share of individuals vaccinated with subsequent doses may gradually decrease.
2. Development and use of Omicron-adapted booster dose before the expected wave. This approach would likely increase the specificity of the responses against the Omicron lineage but also does not come without challenges. Firstly, the Omicron variant continues to evolve, and its novel sublineages, characterized by enhanced transmissibility, are characterized by some unique mutations increasing antibody evasion [79,80]. The question remains which variant of spike protein should be selected as an antigen for such a booster vaccine. Furthermore, studies show that despite the Omicron dominance, other SARS-CoV-2 variants, including Delta, remain in cryptic circulation [81]. If one considers the asymmetric cross-immunization in which a person with a history of Omicron infection is four-fold less protected from Delta infection than protection from Omicron in a Delta-immunized individual [82,83], basing booster strategy on the vaccine adapted only to the Omicron variant could bring the potential risk of contracting other SARS-CoV-2 variants.
3. Development and use of multiple antigen-based (multivariant-adapted) booster dose before the expected wave. This approach would possibly allow inducing a broad immunity against various variants, including Delta, Omicron, Beta, and others. This approach is used against influenza, with trivalent and quadrivalent vaccines targeting three and four strains of the virus, respectively [84]. However, it also comes with some shortcomings. Firstly, the chemical inactivation of SARS-CoV-2 has been shown to induce a transformation of prefusion conformation of spike protein to form resembling postfusion conformation, which is less immunogenic [85]. This challenge can be overcome by developing multivalent subunit vaccines, but their production is longer and more expensive [86]. A more cost- and time-efficient approach would involve the development of multivariant mRNA vaccines. However, these vaccines would require using more than one mRNA molecule to encode different versions of the spike protein. Whether using multiple mRNA molecules in a single-dose vaccine would affect translation efficiency, immunogenicity, and efficacy remain to be understood. There is, however, some evidence that such an approach may provide a broad neutralizing immunity against different SARS-CoV-2 variants, as shown in vivo for mRNA-1273.211 comprising a 1:1 mix of mRNA-1273 (present in the first-generation vaccine developed by Moderna, USA) and mRNA-1273.351 (adapted to Beta variant) [87,88]. More publicly available data is required to understand whether mRNA vaccines adapted to different SARS-CoV-2 variants, including Omicron, are providing efficient protection.
4. Development of vaccines providing broad immune responses with enhanced durability. The main challenge of currently available COVID-19 vaccines is related to a gradual decrease of antibody levels observed within a few months from dose administration [16]. Although a booster dose temporarily restores antibody levels and strengthens cellular responses [89], the provided protection from different outcomes (including infections and hospital admission) of Omicron infection starts to wane after three-four months from administration [44,48,66]. It becomes more and more evident that vaccine strategies that would increase the durability of protection are necessary. This requires more studies to understand which amino acid substitutions could extend the half-life of antibodies but not decrease their neutralization activities and then design an antigen that would trigger their production. Moreover, some promise is also brought with vaccine candidates based on self-amplifying RNAs (saRNA), which enhance antigen presentation and may therefore mount a robust adaptive immune response against SARS-CoV-2 [90,91]. Further studies are required to understand whether saRNA can enhance the durability of protection.

## Figures and Tables

**Table 1:** Summary of studies evaluating the Omicron-neutralizing activity of the first COVID-19 vaccine booster.

Reference	Design	Findings
[33]	A longitudinal study (USA), 85 nursing home residents, 48 healthcare workers. The effect of BNT162b2 booster on humoral immunity and Omicron-specific neutralizing activity were studied. Samples taken after the initial vaccination series, before and 2 weeks after booster vaccination. Neutralization titers against the ancestral and Omicron variants were analyzed.	≥86% of subjects receiving the booster showed detectable Omicron neutralizing activity, compared to 28% after the primary vaccination course. The geometric mean titer values of Omicron-specific neutralization increased by 5.5 fold in nursing home residents. BNT162b2 booster vaccination significantly increased the neutralization levels against the Omicron variant.
[34]	A small US immunogenicity study (20 healthy adults) evaluating the Omicron neutralization after primary course of mRNA-1273 vaccine and booster.	mRNA-1273 booster vaccination increased neutralization titers against the Omicron variant by 20 folds compared to the second vaccine dose.
[35]	A cohort Chinese study with healthcare professionals (n = 77) between 8–14 November 2021. The spike-specific IgG and IgA responses and neutralization activities against ancestral, Delta and Omicron variants were evaluated in subject’s booster with CoronaVac after 9 months from the primary course of vaccination.	>55% of subjects had the Omicron-neutralizing activities after the booster dose. The geometric mean titer increased by 2 fold. The booster of CoronaVac elicited broad and potent adaptive immune responses against Omicron variant.
[38]	A subject-blinded, randomized-controlled trial study in Singapore evaluating reactogenicity and immunogenicity of different COVID-19 vaccine booster combinations. 100 BNT162b2-vaccinated individuals were enrolled and received either homologous (BNT162b2 + BNT162b2 + BNT162b2; ‘BBB’) or heterologous mRNA booster vaccine (BNT162b2 + BNT162b2 + mRNA-1273; ‘BBM’)	At day 28 post-booster, the inhibition percent against the Omicron variant in the BBM group (84.3%) remained significantly higher than in the BBB group (72.8%, *p* = 0.007). Compared to the homologous BNT123b2, the heterologous mRNA-1273 booster vaccination induced a more robust neutralizing response against the Omicron variant in older individuals.
[39]	Samples from healthy adults in China who received a third boosting vaccination with either an inactivated whole-virion vaccine (BBIBP-CorV, homologous booster group) or a protein subunit vaccine (ZF2001, heterologous booster group) after previous priming vaccination by two doses of BBIBP-CorV vaccine. The titers of neutralizing antibodies against the ancestral and Omicron viruses were analyzed.	The booster improved the neutralization titer against Omicron by 4 fold. No difference in neutralization titers against Omicron between the homologous and heterologous groups.
[40]	A phase 4, a non-inferiority, single-blind, randomized study in Brazil. 1240 participants randomly assigned to receive one of four different booster vaccines of either heterologous dosing with ChAdOx1 nCoV-19, BNT162b2, or Ad26.COV2-S, or homologous dosing with CoronaVac. Anti-spike, receptor binding domain, and nucleocapsid responses, and antibody neutralization titers at the 28 days after the booster dose were evaluated.	On day 28, after the booster, all groups had a substantial rise in antibody concentrations. The geometric fold-rise from baseline to day 28 was 77, 152, 90, and 12 for Ad26.COV2-S, BNT162b2, ChAdOx1 nCoV-19, and CoronaVac, respectively. Participants receiving a third heterologous dose had a higher neutralizing response than those receiving the homologous booster.

**Table 2:** Summary of studies evaluating the effectiveness of the first COVID-19 vaccine booster against the Omicron variant.

Reference	Design	Findings
[43]	A large, prospective observational study including data of 11,690 adults across 21 U.S. hospitals. The effectiveness of mRNA vaccination (BT162b2 and mRNA-1273) against Omicron, Delta, and Alpha SARS-CoV-2 was evaluated.	The effectiveness of the mRNA vaccination against the Omicron variant was 65% and 86% for two doses and booster, respectively.
[44]	A test-negative case-control study based on an analysis of 16,063 hospital admissions and 19,699 emergency department admissions across Kaiser Permanente Southern California, a large integrated healthcare system in California, USA, from 1 December 2021 to 6 February 2022. Vaccine effectiveness was calculated in patients aged ≥18 years admitted to a hospital or an emergency department.	The effectiveness of two doses of the BNT162b2 vaccine against the Omicron variant was 41% and 31% against hospital admission and emergency department admission at ≥ 9 months after the second dose, respectively. The effectiveness of BNT162b2 booster against hospital admission due to the Omicron was 85% at < 3 months but fell to 55% at ≥3 months.
[45]	A test-negative case-control design to estimate vaccine effectiveness against symptomatic disease caused by the Omicron and Delta variants in England. Vaccine effectiveness was evaluated after primary immunization with two doses of BNT162b2, ChAdOx1 nCoV-19, or mRNA-1273 vaccine and after a booster dose of BNT162b2, ChAdOx1 nCoV-19, or mRNA-1273. A total of 886,774 persons with symptomatic disease infected with the Omicron variant were identified during the study period.	The increase in the vaccine effectiveness (67.2%, at 2-4 weeks) of the BNT162b2 booster against the Omicron variant was observed among patients who received full-dose BNT162b2 vaccination, and the vaccine effectiveness declined to 45.7% at ≥10 weeks.
[46]	A large, diverse, test-negative case-control study in the USA to evaluate mRNA-1273 vaccine effectiveness against infection and hospitalization with Omicron or Delta. This study included 26,683 SARS-CoV-2 test-positive cases with variants and 109,662 controls.	The effectiveness of mRNA-1273 booster against Omicron infection was 71.6% at 14–60 days after the booster. It decreased to 47.4% at >60 days whereas the vaccine effectiveness of two-dose mRNA-1273 against Omicron infection was only 44% at 14–90 days and waned to 5.9% at 271–365 days.
[47]	A Cox proportional hazards model and a logistic regression analysis based on individual-level population-wide data from the Czech Republic to estimate risks of infection and hospitalization, including severe COVID-19. This study evaluated the protection due to vaccination or previous SARS-CoV-2 infection against COVID-19 infection, hospital admission, oxygen therapy and intensive care unit admission.	The vaccine effectiveness of a recent (≤2 months) initial vaccination against Omicron and Delta infections was 43% and 73%, respectively. The booster dose increased effectiveness against infection to 56% (Omicron) and 90% (Delta). The effectiveness against Omicron-related hospitalization of initial vaccination and booster was 45% and 87%, respectively.
[48]	Multistate analysis of 241,204 emergency department/urgent care encounters and 93,408 hospitalizations among adults with COVID-19–like illness during 26 August 2021–22 January 2022 in the USA. This study evaluated the durability of protection after 3 doses during periods of Delta or Omicron variant predominance in the USA.	During the 2 months after the booster (third dose), the vaccine effectiveness value of the booster against COVID-19-associated ED/UC visits and hospitalizations was 87% and 91%, respectively. But by the fourth month after the booster vaccination, the vaccine effectiveness value decreased to 66% and 78%, respectively.
[49]	A matched, retrospective cohort study design that emulated a target trial in the USA. This study evaluated the relative vaccine effectiveness (RVE) of a homologous booster dose of mRNA vaccine compared with the primary vaccine series alone in preventing infection, hospitalization, and intensive care unit admission, and death in the Department of Veterans Affairs healthcare system in the USA. Booster group: 198,860 subjects receiving BNT162b2 mRNA booster and 264,090 receiving mRNA-1273. No-booster group: 198,860 subjects receiving BNT162b2 mRNA booster and 264,090 receiving mRNA-1273.	The RVE of the booster for confirmed infection, hospitalization, and intensive care unit admission or death was 19%, 52%, and 83%, respectively. RVE was highest for subjects receiving the booster vaccine within 28 days of the starting period of Omicron predominance (40% for BNT162b2 and 30% for mRNA-1273), The protection against infection was negligible for both vaccines for subjects with ≥4 months since booster receipt. Despite the low RVE of mRNA booster vaccine dose in preventing Omicron infection, it was substantial in preventing hospitalization.
[56]	A test-negative, case–control analysis to assess the effectiveness of four vaccination regimens against symptomatic infection with severe acute respiratory syndrome coronavirus 2 during the Omicron predominance period in the USA. Four regimens included a single priming dose of Ad26.COV2.S, a single priming dose of Ad26.COV2.S plus a booster dose of Ad26.COV2.S (Ad26.COV2.S/Ad26.COV2.S), a single priming dose of Ad26.COV2.S plus a booster dose of mRNA vaccine (Ad26.COV2.S/mRNA), and two priming doses of an mRNA vaccine plus a booster dose of mRNA vaccine (mRNA/mRNA/mRNA). Either the BNT162b2 vaccine (Pfizer–BioNTech) or the mRNA-1273 vaccine (Moderna) was used in the mRNA vaccine regimens.	Compared with no vaccination, the vaccine effectiveness of the Ad26.COV2.S regimen against symptomatic infection during the period of 14 days to 1 month since receipt of the last dose and during the period of 2 to 4 months since receipt of the last dose was 17.8% and 8.4%, respectively. During the studied periods, the vaccine effectiveness of Ad26.COV2.S/Ad26.COV2.S regimen were 27.9% and 29.2%, respectively; 61.3% and 54.3% for the Ad26.COV2.S/mRNA regimen, respectively; 68.9% and 62.8% for the mRNA/mRNA/mRNA regimen, respectively. All three booster regimens protected against symptomatic infection. The highest vaccine effectiveness was in the regimens including mRNA vaccine booster dose, the lowest in the homologous group.
[57]	Two matched retrospective cohort studies to assess the booster effectiveness of booster, compared to that of initial vaccination conducted during the Omicron infection period (19 December 2021–26 January 2022) in Qatar. In a population of 2,239,193 persons receiving at least two doses of BNT162b2 or mRNA-1273 vaccine, those receiving a booster were matched with persons not receiving a booster.	The effectiveness of the BNT162b2 and mRNA-1273 booster against symptomatic omicron infection was 49.4% and 47.3%, respectively. The mRNA boosters were highly effective against Delta infection, but less effective against Omicron infection. mRNA boosters conferred strong protection against Delta and Omicron-related hospitalization and death.
[58]	A test-negative design using consolidated national administrative data in Malaysia to compare the vaccine effectiveness of homologous and heterologous BNT162b2, CoronaVac, and AZD1222 booster vaccination against SARS-CoV-2 infection during the Delta and Omicron predominance periods (27 October 2021–4 February 2022; and 5–22 February 2022, respectively).	Compared to the primary vaccination reference (2× BNT162b2), the adjusted marginal vaccine effectiveness of six booster vaccination regimens ((1). PPP (3× BNT162b2), (2). SSA (2× CoronaVac + AZD1222), (3). SSP (2× CoronaVac+BNT162b2); (4). SSS (3× CoronaVac), (5). AAP (2× AZD1222+BNT162b2), and (6) AAA (3× AZD1222)) were 51.08%, 49.05%, 47.64%, 33.42%, 52.96%, 30.14%, respectively. The heterologous booster vaccinations were associated with higher adjusted marginal effectiveness. A BNT162b2 booster was recommended for subjects receiving the primary regimen with inactivated and vectored primary vaccines.
[59]	A test-negative design study to estimate vaccine effectiveness of homologous and heterologous COVID-19 booster following 1 Ad.26.COV2.S vaccine dose against COVID-19-associated emergency department and urgent Care (EDUC) encounters and hospitalizations among adults across 10 USA states during the Omicron predominance period (December 2021–March 2022). This study included 80,287 emergency department/urgent care encounters and 25,244 hospitalizations.	The vaccine effectiveness of heterologous booster practice (1 × Ad26.COV2.S/1 × mRNA dose) against emergency department/urgent care visits and hospitalization was 79% and 78%, respectively, higher than in the homologous Ad26.COV2.booster vaccination (54% and 67%, respectively). The subjects receiving Ad.26.COV2.S primary vaccine should preferentially receive the mRNA vaccine booster to improve the protection against Omicron.

**Table 3:** Summary of studies evaluating immunogenicity and efficacy/effectiveness of the second booster of COVID-19 vaccine.

Reference	Design	Findings
[67]	Prospective, open-label, non-randomized study Participants–healthcare workers ≥ 18Arms treatment arm 3 doses BNT162b2 + 4th dose of BNT162b2 (*n* = 154) or mRNA-1273 (*n* = 120)age-matched control arm 3 doses BNT162b2 (*n* = 547)	Immunogenicity and efficacy 9–10-fold increase in IgG RBD and neutralizing antibody titers 2 weeks after the 4th dose of both formulations. 8–10-fold increase in live neutralization against Omicron and other variants with titer restoration to the peak after the 3rd dose of BNT162b2. Efficacy against any SARS-CoV-2 infection: 30% for BNT162b2, 11% for mRNA-1273 compared to controls. Percentage of Omicron infection: 18.3% for BNT162b2, 20.7% for mRNA-1273 and 25% in control arm. Protection against symptomatic disease: 43% for BNT162b2, 31% for mRNA-1273 compared to controls.
[68]	Retrospective real-world population-based study Participants ≥ 60 yrs four-dose group (8–14 days after 4th dose of BNT162b2) internal control four-dose group (3–5 days after 4th dose of BNT162b2) control three-dose group (persons after 3 doses of BNT162b2, eligible for 4th dose, but not yet vaccinated)	Effectiveness The adjusted rate of severe COVID-19 in the fourth week after receipt of the 4th dose was lower than that in the three-dose group by a factor of 3.5 (95% confidence interval [CI], 2.7 to 4.6) and lower than that in the internal control group by a factor of 2.3 (95% CI, 1.7 to 3.3). Protection against severe disease did not wane during the 6 weeks after receipt of the 4th dose. The adjusted rate of confirmed infection in the fourth week after receipt of the 4th dose was lower than that in the three-dose group by a factor of 2.0 (95% CI, 1.9 to 2.1) and lower than that in the internal control group by a factor of 1.8 (95% CI, 1.7 to 1.9); this protection waned in later weeks.
[70]	Retrospective real-world population-based study Participants ≥ 60 yrs four-dose group (after 4th dose of BNT162b2) Control three-dose group (persons after 3 doses of BNT162b2, eligible for 4th dose, but not yet vaccinated)	Effectiveness–protection assessed 7–30 days and 14–30 days after 4th dose against SARS-CoV-2 infection confirmed by RT-PCR–45% and 52% Symptomatic COVID-19–55% and 61% COVID-19-related hospitalization–68% and 72% Severe COVID-19–62% and 64% COVID-19-related death–74% and 76%

## Data Availability

Not applicable.
